# Receiving three doses of inactivated or mRNA COVID-19 vaccines was associated with lower odds of long COVID symptoms among people with a history of SARS-CoV-2 infection in Hong Kong, China: a cross-sectional survey study

**DOI:** 10.1017/S0950268824001687

**Published:** 2024-12-11

**Authors:** Chen Zheng, Fuk-yuen Yu, Paul Shing-fong Chan, Fenghua Sun, Xiang-Ke Chen, Wendy Ya-Jun Huang, Stephen Heung-Sang Wong, Yuan Fang, Zixin Wang

**Affiliations:** 1 Department of Health and Physical Education, Faculty of Liberal Arts and Social Sciences, The Education University of Hong Kong, Hong Kong, China; 2 Centre for Health Behaviours Research, JC School of Public Health and Primary Care, The Chinese University of Hong Kong, Hong Kong, China; 3 Division of Life Science, School of Science, The Hong Kong University of Science and Technology, Hong Kong, China; 4 Academy of Wellness and Human Development, Hong Kong Baptist University, Hong Kong, China; 5 Dr Stephen Hui Research Centre for Physical Recreation and Wellness, Hong Kong Baptist University, Hong Kong, China; 6 Department of Sports Science and Physical Education, Faculty of Education, The Chinese University of Hong Kong, Hong Kong, China

**Keywords:** booster, inactivated vaccines, Long COVID, mRNA vaccines, post-acute sequelae of COVID-19, post-COVID-19 condition, vaccination

## Abstract

High prevalence of long COVID symptoms has emerged as a significant public health concern. This study investigated the associations between three doses of COVID-19 vaccines and the presence of any and ≥3 types of long COVID symptoms among people with a history of SARS-CoV-2 infection in Hong Kong, China. This is a secondary analysis of a cross-sectional online survey among Hong Kong adult residents conducted between June and August 2022. This analysis was based on a sub-sample of 1,542 participants with confirmed SARS-CoV-2 infection during the fifth wave of COVID-19 outbreak in Hong Kong (December 2021 to April 2022). Among the participants, 40.9% and 16.1% self-reported having any and ≥3 types of long COVID symptoms, respectively. After adjusting for significant variables related to sociodemographic characteristics, health conditions and lifestyles, and SARS-CoV-2 infection, receiving at least three doses of COVID-19 vaccines was associated with lower odds of reporting any long COVID symptoms comparing to receiving two doses (adjusted odds ratio [AOR]: 0.69, 95% CI: 0.54, 0.87, *P* = .002). Three doses of inactivated and mRNA vaccines had similar protective effects against long COVID symptoms. It is important to strengthen the coverage of COVID-19 vaccination booster doses, even in the post-pandemic era.

## Introduction

As of 19 May 2024, the global number of coronavirus disease 2019 (COVID-19) cases had reached 775 million, with a total reported mortality of about 7 million worldwide [1]. In Hong Kong, the study site, more than 3 million people were infected with SARS-CoV-2 with a cumulative death of 9,287 recorded [2,3]. Albeit people may fully recover from the infection, some people may be prone to the sequelae of COVID-19 by experiencing long COVID symptoms [4].

Long COVID, or post-COVID-19 condition that proposed by the World Health Organization (WHO), occurs in people with history of SARS-CoV-2 infection, which the symptoms continue or develop three months after the onset of SARS-CoV-2 infection and last for at least two months that cannot be explained by other diagnosis [5]. However, there was a lack of consensus in defining long COVID conditions in studies conducted prior to the issuance of WHO definition, which created challenges to estimate the overall prevalence of such conditions. A meta-analysis estimated the global pooled prevalence of post-COVID-19 conditions, which was defined as having any symptoms or at least one new or persisting symptom at least 28 days after infection [4]. The results showed that the global pooled prevalence of post-COVID-19 conditions was about 50% for hospitalized and 34% for non-hospitalized COVID-19 survivors [4]. However, such prevalence might be overestimated as asymptomatic individuals were not reflected in most included studies of the aforementioned meta-analysis. Moreover, included studies conducted in early 2020 mainly focussed on older and higher-risk individuals and might not reflect the situation in general population [4]. In Hong Kong, 55% of patients reported having any long COVID symptom after infection with the Omicron variant in a study adhered to the WHO definition, which the study investigated the prevalence of long COVID among non-hospitalized patients with confirmed SARS-CoV-2 infection that received consultation services provided by a Chinese medicine research institute [6]. Some people who contracted SARS-CoV-2 may be prone to experience multiple long COVID symptoms for more than six months [7], the multi-manifestations of symptoms across organs led this condition to also be coined as post-COVID syndrome [8]. Fatigue, cognitive symptoms (e.g., brain fog), breathlessness or dyspnoea, muscle pain or myalgia, and sleep problems were the most common long COVID symptoms observed across review studies [4,9]. Long COVID symptoms may induce detrimental or even devastating effects to daily functioning and employment [10], mental health [11], perceived physical health [10,11], and health-related quality of life [9]. Furthermore, studies showed that long COVID was associated with a substantial increase in utilization of healthcare services (both inpatient and outpatient) and economic costs [12].

Previous studies have identified some factors associated with the development of long COVID symptoms. A positive association between severity of SARS-CoV-2 infection during the acute phase and long COVID was observed [13]. Patients with more than five symptoms during the initial SARS-CoV-2 infection and those that required hospital admission were more likely to develop long COVID [14]. Pre-existence of certain chronic health conditions, such as asthma, diabetes, heart disease, and neurocognitive and mental health conditions at the onset of SARS-CoV-2 infection were also significantly associated with long COVID [14,15]. Additionally, some demographic characteristics were associated with long COVID. For example, studies showed that females and older age were found to be more likely to exhibit long COVID [4]. These factors were examined in the current study.

COVID-19 vaccination is one of the most cost-effective measures to prevent infection, deaths, and other severe consequences caused by COVID-19 [16,17]. Two underlying mechanisms have been suggested for the association between taking up COVID-19 vaccination booster doses and reduced odds of long COVID. First, as booster doses are effective in reducing the severity of COVID-19 infection, this may then translate into a lower risk of developing organ or systemic derangements, and thus reduce symptoms onset and duration which consequently result in lower odds of long COVID [18]. Second, booster doses may accelerate clearance of the remaining SARS-CoV-2 in the human body or could also reduce the exaggerated inflammatory and/or immune response associated with long COVID development [18,19]. In Hong Kong, two types of COVID-19 vaccines are available, i.e., the inactivated and mRNA vaccines [20]. A systematic review identified 12 studies reporting the associations between vaccination status before SARS-CoV-2 infection and the presence of any long COVID symptoms [21]. These studies focussed on the effects of mRNA (Pfizer or Moderna) or viral vector vaccines (Janssen or AstraZeneca) [21]. The odds ratio (OR) of developing long COVID with one dose of vaccine ranged from 0.22 to 1.03, and 0.25 to 1.00 with two doses [21]. Only one study reported that three doses of mRNA COVID-19 vaccines could significantly reduce the risk of long COVID (OR: 0.16) [21]. Another study reported that taking up two doses of any COVID-19 vaccines, including mRNA, viral vector, and inactivated vaccines, was associated with lower odds of any long COVID [22]. However, the aforementioned study did not specify the protective effects conferred by different types of COVID-19 vaccines [22]. To our knowledge, there was a lack of studies investigating the protective effects of three doses of inactivated COVID-19 vaccines on long COVID. Moreover, no study compared the effectiveness of three doses of mRNA vaccines with three doses of inactivated vaccines in reducing the odds of long COVID.

To address the aforementioned knowledge gaps, this study aimed at examining the associations between three doses of COVID-19 vaccination (mRNA and inactivated vaccine) and (1) having any of the long COVID symptoms and (2) ≥3 types of long COVID symptoms among people with a history of SARS-CoV-2 infection in Hong Kong, China.

## Methods

### Study design

This study is a secondary analysis of a cross-sectional online survey investigating the associations between sedentary lifestyles and the risk of acute and post-acute COVID-19 sequelae among residents in Hong Kong between June and August 2022 [23]. The fifth wave of the outbreak of COVID-19 that dominated by the Omicron variant occurred between December 2021 and April 2022 in Hong Kong. The original study was launched approximately three months after the peak of the wave. We extracted a sub-sample of participants who self-reported having confirmed SARS-CoV-2 infection during the fifth wave of the COVID-19 outbreak from the original study for current investigation.

### Participants and data collection

Inclusion criteria of the original online survey were (1) adults aged 18 years or above, (2) currently living in Hong Kong, (3) able to read traditional Chinese or English, and (4) with internet access. The original online survey, which consist of 243 questions in Chinese and English that took about 20 mins to complete, was developed by using Qualtrics with the advertisement and survey link distributed through social media, websites, e-mails, online groups, posters, and leaflets. In the advertisement of the original online survey, the aim towards investigating the associations between sedentary lifestyles and symptoms or sequelae of COVID-19 during the fifth wave of COVID-19 in Hong Kong was delineated, and people could participate in the survey regardless of contracting with SARS-CoV-2 or not. Before starting the online survey, participants read a statement that participation was voluntary and their personal information would be kept confidential. Digital informed consent was obtained before participants could start answering the online survey. The online survey platform checked for completeness before submission, allowing participants to review and change their responses. No incentives were given to participants. The data were stored on the survey platform server and protected by password. Only the principal investigator of this project had the access to the database. During the study period, a total of 7,089 people accessed the original online survey, 87 did not provide consent to join the study, 60 were ineligible (not living in Hong Kong or under 18 years old), 2092 did not complete the survey, and 4,850 completed the online survey. We extracted a sub-sample of 1,542 participants, who self-reported having confirmed SARS-CoV-2 infection (either by nucleic acid amplification tests [NAAT] or rapid antigen testing [RAT]) during the fifth wave of outbreak (between December 2021 and April 2022) for the analysis. The original study was conducted in accordance with the principles of Declaration of Helsinki and approved by the Survey and Behavioural Research Ethics Committee, The Chinese University of Hong Kong (SBRE-21-0762).

### Measures

Sociodemographic characteristics (age, sex assigned at birth, ethnicity, relationship status, personal monthly income, education level, employment status, and living alone or not) and health conditions and lifestyles (e.g. body mass index, current smoking and drinking status, presence of chronic health conditions, history of organ transplantation) were collected. Participants also reported hospital admission and intensive care unit admission due to SARS-CoV-2 infection during the fifth wave of COVID-19 outbreak in Hong Kong.

Self-reported long COVID symptoms were measured with reference to the definition proposed by the WHO [5]. First, participants were asked “Did you experience the following symptoms after SARS-CoV-2 infection or during the period between March and June 2022? If you experienced such symptoms before SARS-CoV-2 infection, please select no”. Participants were asked to indicate from a checklist of 14 common post-COVID-19 conditions, which included: (1) fatigue, (2) shortness of breath, (3) chest pain or tightness, (4) problems with memory and concentration (brain fog), (5) difficulty to fall asleep, (6) heart palpitations, (7) dizziness, (8) pins and needles, (9) joint pain, (10) feeling depressed or anxious, (11) persistent cough, (12) muscle pain, (13) loss of smell or taste, and (14) fever with response categories of 1 = Yes and 2 = No. Following each of the aforementioned symptoms, a question on tapping the duration of the symptom (1 = less than one week, 2 = 1 week to less than 1 month, 3 = 1 to 2 months, 4 = more than 2 months) was asked. Participants who experienced any of these symptoms for more than 2 months after SARS-CoV-2 infection or during the period between March and June 2022 were considered exhibiting long COVID. Owing to the multi-manifestation nature of long COVID symptoms across organs, the presence of ≥3 types of long COVID symptoms was also measured apart from exhibiting any of the 14 common long COVID symptoms.

COVID-19 vaccination status was measured by a number of doses and types of COVID-19 vaccines received prior to the fifth wave of the COVID-19 outbreak. In Hong Kong, an inactivated COVID-19 vaccine (SinoVac CronaVac) and mRNA vaccine (Pfizer-BioNTech) were available. During the time when this study was conducted, adults in Hong Kong were required to present vaccination records before entering public spaces. The vaccination pass contained information about number of doses, vaccine type, and date of COVID-19 vaccines received by the users.

Information related to SARS-CoV-2 infection was also collected. Participants were asked if they were infected with SARS-CoV-2 for the first time during the fifth wave of the COVID-19 outbreak in Hong Kong (1 = yes, 2 = no, 3 = unknown) and methods for confirming infection (NAAT, RAT, or both). The overall severity of SARS-CoV-2 infection was gauged by asking them to rate the severity with the response categories: 1 = asymptomatic (no symptoms), 2 = mild (with common symptoms, but without shortness of breath, dyspnoea, or abnormal chest imaging), 3 = moderate (with shortness of breath or severe cough), 4 = severe (with dyspnoea or abnormal chest imaging), and 5 = critical (with respiratory failure, septic shock, or multiple organ dysfunctions).

### Statistical analysis

Descriptive statistics towards frequencies and percentages were used to present all study variables. Using having any self-reported long COVID symptoms and self-reported ≥3 types of long COVID symptoms as the dependent variables, univariate logistic regression models were employed to measure the associations between outcome variables and sociodemographic characteristics, health conditions and lifestyles, and SARS-CoV-2 infection with OR and respective 95% confidence interval (CI) reported. Then, a single multivariate logistic regression model was fitted involving COVID-19 vaccination status and all significant variables related to sociodemographic characteristics, health conditions and lifestyles, and SARS-CoV-2 infection for obtaining the adjusted odds ratio (AOR) with 95% CI reported. In addition, multivariate logistic regression models were conducted to compare the effects of three doses of inactivated vaccines with three doses of mRNA vaccines (reference group) in reducing the odds of any self-reported long COVID symptoms and self-reported ≥3 types of long COVID symptoms, after adjusting for all significant variables related to sociodemographic characteristics, health conditions and lifestyles, and COVID-19 infection. All analyses were conducted using SPSS (version 28; IBM Corp., Armonk, NY, USA) with *P* < 0.05 considered as statistically significant.

## Results

### Sociodemographic characteristics, health conditions and lifestyles, and SARS-CoV-2 infection

Approximately half of the participants were aged 18–30 years (51.3%) and had a normal range of BMI (18.5–22.9) (52.0%). About two-thirds were females (63.2%), currently single (68.5%), and had a personal monthly income higher than HK $13400 (US $1717.9) (65.4%). The majority were Chinese (99.4%), full-time employed (82.4%), not current smokers (95.1%), not binge drinkers (95.5%), attained tertiary education or above (79.2%), living with other household member (92.0%), and without a history of organ transplantation (99.9%). Less than 10% had any of the chronic conditions (8.4%).

The date of receiving confirmed SARS-CoV-2 infection during the fifth wave of the COVID-19 outbreak was between 3 January 2022 and 29 April 2022. Only 14 (0.9%) participants were hospitalized, and none of them were admitted to the ICU due to SARS-CoV-2 infection. The majority of participants were infected with SARS-CoV-2 for the first time (97.9%) and had mild symptoms (76.2%). Approximately half of them used RAT to confirm SARS-CoV-2 infection (56.7%). (Table 1).

**Table 1.** Background characteristics of the participants (*n* = 1,542)

	*n*	%
**Sociodemographic characteristics**		
Age group, years		
18–30	791	51.3
31–40	345	22.4
41–50	207	13.4
51–60	127	8.2
>60	72	4.7
Sex assigned at birth		
Male	567	36.8
Female	975	63.2
Ethnicity		
Chinese	1,532	99.4
Non-Chinese	10	0.6
Relationship status		
Single/divorced/widowed	1,057	68.5
Married with a partner	485	31.5
Personal monthly income, HK$ (US$)		
<13,400 (1717.9)	534	34.6
13,400–29,500 (1717.9–3,782.1)	604	39.2
> 29,500 (3,782.1)	404	26.2
Education level		
Primary or below	273	17.7
Secondary	48	3.1
Tertiary and above	1,221	79.2
Full-time employment		
No	272	17.6
Yes	1,270	82.4
Living alone		
No	1,418	92.0
Yes	124	8.0
**Health conditions and lifestyles**		
BMI		
Underweight (<18.5)	169	11.0
Normal range (18.5–22.9)	802	52.0
Overweight (23–24.9)	267	17.3
Obesity (≥25)	304	19.7
Current smokers		
No	1,467	95.1
Yes	75	4.9
Binge drinking		
No	1,473	95.5
Yes	69	4.5
Presence of chronic conditions, Yes		
Hypertension	87	5.6
Diabetes	43	2.8
Coronary heart diseases	4	0.3
Cerebrovascular diseases	7	0.5
Chronic kidney diseases	5	0.3
Cancers	15	1.0
Neurodegenerative disorders	0	0
Chronic obstructive pulmonary disease (COPD)	2	0.1
HIV infection	1	0.1
Any of above	130	8.4
History of organ transplantation		
No	1,541	99.9
Yes	1	0.1
**Information about COVID–19 infection during the fifth wave of COVID–19 outbreak in Hong Kong**		
Infected with COVID–19 for the first time		
Yes	1,509	97.9
No	33	2.1
Methods to confirm COVID–19 infection		
Nucleic acid amplification tests	256	16.6
Rapid antigen tests	875	56.7
Both	411	26.7
Overall severity of COVID–19 infection		
Asymptomatic	72	4.7
Mild (with common symptoms, but without shortness of breath, dyspnoea, or abnormal chest imaging)	1,175	76.2
Moderate (with shortness of breath or severe cough)	281	18.2
Severe (with dyspnoea or abnormal chest imaging)	14	0.9
Critical (with respiratory failure, septic shock, or multiple organ dysfunctions)	0	0
Hospital admission due to COVID–19 infection		
No	1,528	99.1
Yes	14	0.9
Intensive care unit (ICU) admission due to COVID–19 infection		
No	1,542	100
Yes	0	0
**Self-reported long COVID symptoms, yes**		
Fatigue	315	20.4
Shortness of breath	108	7.0
Chest pain or tightness	66	4.3
Problems with memory and concentration (brain fog)	400	25.9
Difficult to fall asleep	175	11.3
Heart palpitations	80	5.2
Dizziness	81	5.3
Pins and needles	46	3.0
Joint pain	85	5.5
Feeling depressed or anxious	131	8.5
Persistent cough	88	5.7
Muscle pain	72	4.7
Loss of smell or taste	18	1.2
Fever	6	0.4
Any of above	631	40.9
Presence of ≥3 symptoms	249	16.1
**COVID–19 vaccination status**		
Doses of COVID–19 vaccines received by the participants prior to the fifth wave of COVID–19 outbreak in Hong Kong		
2 doses	888	57.6
3–4 doses	491	31.8
0–1 dose	163	10.6

### Self-reported long COVID symptoms

The prevalence of any long COVID symptoms and ≥ 3 types of long COVID symptoms was 40.9% and 16.1% respectively. The most commonly reported long COVID-19 symptoms were brain fog (25.9%), followed by fatigue (20.4%), difficulty to fall asleep (11.3%), and feeling depressed or anxious (8.5%). (Table 1).

### COVID-19 vaccination status

Among the participants, 1,379 (89.4%) of them received at least two doses of COVID-19 vaccination. Among 491 (31.8% of all respondents) participants who received at least three doses of COVID-19 vaccination, the most common choice was three doses of Pfizer-BioNTech (*n* = 345), followed by three doses of SinoVac CoronaVac (*n* = 98), two doses of SinoVac CoronaVac plus one dose of Pfizer-BioNTech (*n* = 32), and two doses of Pfizer-BioNTech plus one dose of SinoVac CoronaVac (*n* = 11) (Table 1 and Table S1 in Appendix 1).

### Factors associated with self-reported having any long COVID symptoms

Older age (31–40 years: OR: 1.61, 95% CI: 1.24, 2.08, *P* < .001; 41–50 years: OR: 1.92, 95% CI: 1.41, 2.62, *P* < .001; reference: 18–31 years), being female (OR: 1.35, 95% CI: 1.10, 1.68, *P* = .01), and having moderate-to-severe symptoms following SARS-CoV-2 infection (OR: 3.54, 95% CI: 2.00, 6.28, *P* < .001; reference: asymptomatic) were associated with higher odds of having any long COVID-19 symptoms (Table 2). After adjusting for these significant variables, receiving at least three doses of COVID-19 vaccination was associated with lower odds of having any long COVID symptoms (AOR: 0.69, 95% CI: 0.54, 0.87, *P* = .002; reference group: receiving 2 doses). (Table 3).

**Table 2.** Associations between background characteristics and self-reported long COVID symptoms

	Self-reported having any long COVID symptoms			Self-reported having ≥3 types of long COVID symptoms		
	*n*/N	OR (95% CI)	*P* values	*n*/N	OR (95% CI)	*P* values
**Sociodemographic characteristics**						
Age group, years						
18–30	283/791	1.0		92/791	1.0	
31–40	163/345	1.61 (1.24–2.08)	<0.001	72/345	2.00 (1.43–2.81)	<0.001
41–50	107/207	1.92 (1.41–2.62)	<0.001	52/207	2.55 (1.74–3.74)	<0.001
51–60	53/127	1.29 (0.88–1.88)	0.20	22/127	1.59 (0.96–2.65)	0.07
>60	25/72	0.96 (0.58–1.58)	0.86	11/72	1.37 (0.70–2.70)	0.36
Sex assigned at birth						
Male	206/567	1.0		73/567	1.0	
Female	425/975	1.35 (1.10–1.68)	0.01	176/975	1.49 (1.11–2.00)	0.01
Ethnicity						
Chinese	629/1532	1.0		248/1532	1.0	
Non-Chinese	2/10	0.36 (0.08–1.70)	0.20	1/10	0.58 (0.07–4.56)	0.60
Relationship status						
Single/divorced/widowed	428/1057	1.0		157/1057	1.0	
Married with a partner	203/485	1.06 (0.85–1.32)	0.61	92/485	1.34 (1.01–1.78)	0.04
Personal monthly income, HK$ (US$)						
<13,400 (1717.9)	202/534	1.0		69/534	1.0	
13,400–29,500 (1717.9–3,782.1)	262/604	1.26 (0.99–1.60)	0.06	116/604	1.60 (1.16–2.22)	0.004
>29,501 (3,782.2)	167/404	1.16 (0.89–1.51)	0.28	64/404	1.27 (0.88–1.83)	0.21
Education level						
Primary or below	121/273	1.0		52/273	1.0	
Secondary	17/48	0.69 (0.36–1.30)	0.25	9/48	0.98 (0.45–2.15)	0.96
Tertiary and above	493/1221	0.85 (0.65–1.11)	0.23	188/1221	0.77 (0.55–1.09)	0.14
Full-time employment						
No	109/272	1.0		37/272	1.0	
Yes	522/1270	1.04 (0.80–1.36)	0.75	212/1270	1.27 (0.87–1.86)	0.21
Living alone						
No	579/1418	1.0		227/1418	1.0	
Yes	52/124	1.05 (0.72–1.52)	0.81	22/124	1.13 (0.70–1.83)	0.62
**Health conditions and lifestyles**						
BMI						
Underweight (<18.5)	66/169	1.0		25/169	1.0	
Normal range (18.5–22.9)	308/802	0.97 (0.69–1.37)	0.88	121/802	1.02 (0.64–1.63)	0.92
Overweight (23–24.9)	112/267	1.13 (0.76–1.67)	0.55	41/267	1.05 (0.61–1.79)	0.87
Obesity (≥25)	145/304	1.42 (0.97–2.09)	0.07	62/304	1.48 (0.89–2.45)	0.13
Current smokers						
No	601/1467	1.0		240/1467	1.0	
Yes	30/75	0.96 (0.60–1.54)	0.87	9/75	0.70 (0.34–1.42)	0.32
Binge drinking						
No	607/1473	1.0		240/1473	1.0	
Yes	24/69	0.76 (0.46–1.26)	0.29	9/69	0.77 (0.38–1.57)	0.48
Presence of any chronic conditions						
No	569/1412	1.0		221/1412	1.0	
Yes	62/130	1.35 (0.94–1.94)	0.10	28/130	1.48 (0.95–2.30)	0.08
**Information about COVID–19 infection during the fifth wave of COVID–19 outbreak in Hong Kong**						
Infected with COVID–19 for the first time						
Yes	620/1509	1.0		242/1509	1.0	
No	11/33	0.72 (0.35–1.49)	0.37	7/33	1.41 (0.61–3.28)	0.43
Overall severity of COVID–19 infection						
Asymptomatic	19/72	1.0		8/72	1.0	
Mild	447/1175	1.71 (1.00–2.93)	0.05	158/1175	1.24 (0.59–2.64)	0.57
Moderate to severe	165/295	3.54 (2.00–6.28)	<0.001	83/295	3.13 (1.44–6.82)	<0.001

OR: crude odds ratios.

CI: confidence interval.

**Table 3.** Associations of COVID-19 vaccination status and COVID-19 infection with self-reported long COVID symptoms (*n* = 1,542)

	*n*/N	OR (95% CI)	*P* values	AOR (95% CI)	*P* values
**1. Self-reported having any long COVID symptoms**					
Number of doses of COVID–19 vaccines received by the participants					
2	388/888	1.0		1.0	
3–4	170/491	0.68 (0.54–0.86)	0.001	0.69 (0.54–0.87)	0.002
0–1	73/163	1.05 (0.75–1.46)	0.80	0.97 (0.68–1.36)	0.84
**2. Self-reported having ≥ 3 long COVID symptoms**					
Number of doses of COVID–19 vaccines received by the participants					
2	151/888	1.0		1.0	
3–4	62/491	0.71 (0.51–0.97)	0.03	0.72 (0.51–1.00)	0.051
0–1	36/163	1.38 (0.92–2.08)	0.12	1.26 (0.82–1.92)	0.29

AOR: adjusted odds ratios, odds ratios adjusted for significant variables related to sociodemographic characteristics, health conditions and lifestyles, and COVID-19 infection during the fifth wave of COVID-19 outbreak in Hong Kong listed in Table 2

CI: confidence interval.

### Factors associated with self-reported having ≥ 3 types of long COVID symptoms

As compared to participants who were 18–30 years, those aged 31–40 years (OR: 2.00, 95% CI: 1.43, 2.81, *P* < .001) and 41–50 years (OR: 2.55, 95% CI: 1.74, 3.74, *P* < .001) had higher odds of reporting multiple long COVID symptoms. In addition, being female (OR: 1.49, 95% CI: 1.11, 2.00, *P* = .01), married with a partner (OR: 1.34, 95% CI: 1.01, 1.78, *P* = .04), had a personal monthly income of HK $13400–29,500 (US $1717–3,782.1) (OR: 1.60, 95% CI: 1.16, 2.22, *P* = .004; reference group: < HK $13400), and had moderate-to-severe symptoms following SARS-CoV-2 infection (OR: 3.13, 95% CI: 1.44, 6.82, *P* < .001; reference: asymptomatic) were also associated with higher odds of reporting ≥3 types of long COVID symptoms (Table 2). After adjusting for these significant variables, the association between receiving at least three doses of COVID-19 vaccination and having ≥3 types of long COVID symptoms was not statistically significant (AOR: 0.72, 95% CI: 0.51, 1.00, *P* = .051; reference group: receiving 2 doses). (Table 3).

### Associations between types of COVID-19 vaccination received by the participants and self-reported long COVID symptoms

As compared to those who received two doses of Pfizer-BioNTech, participants who received three doses of Pfizer-BioNTech or SinoVac CoronaVac had lower odds of reporting any long COVID-19 symptoms (three doses of Pfizer-BioNTech: AOR: 0.68, 95% CI: 0.52, 0.90, *P* = .006; three doses of SinoVac CoronaVac: AOR: 0.57, 95% CI: 0.35, 0.90, *P* = .02) (Table S2 in Appendix 1). As compared to those who received three doses of Pfizer-BioNTech, participants who received three doses of SinoVac CoronaVac had similar odds of reporting any long COVID symptoms (AOR: 0.74, 95% CI: 0.44, 1.23, *P* = .25) or ≥ 3 types of long COVID symptoms (AOR: 0.82, 95% CI: 0.40, 1.68; *P* = .59).

## Discussion

This study contributed evidence to the associations between receiving three doses of inactivated COVID-19 vaccines and presence of any and ≥ 3 long COVID symptoms among Chinese people with SARS-CoV-2 infection during the Omicron predominance. This was also one of the first studies to compare the protective effects of three doses of inactivated vaccines and three doses of mRNA vaccines on reducing odds of long COVID symptoms. The findings provided some useful data about how different combinations of primary and booster COVID-19 vaccine doses would affect likelihood of long COVID symptoms. Such findings might have implications on vaccination programme planning in China and other countries that mainly used inactivated and mRNA COVID-19 vaccines.

In this study, 40.9% of participants self-reported having any long COVID symptoms. Such level was lower than the figures observed in mainland China (45–76%) [24–27] and the pooled prevalence in Asia (51%) [4]. One possible reason for the difference might be the age distributions between study samples. Our participants were younger (26.3% were over 40 years old) than studies conducted in mainland China (78.3% were over 40 years) [24,25] and other Asian countries such as India (72% were over 40 years) [28]. Increasing age was consistently shown to be associated with a higher risk of experiencing long COVID [4]. Hong Kong is an ageing society, and the proportion of individuals aged 65 years or above reached 20.5% in 2021 [29]. The risk of SARS-CoV-2 infection was higher among older adults as compared to their younger counterparts during the fifth wave of COVID-19 outbreak in Hong Kong [30]. Therefore, our findings could only reflect the situations among younger adults in Hong Kong. Future studies are needed to understand the levels of long COVID symptoms among older adults.

Our findings also highlighted the role of COVID-19 vaccination booster dose in reducing risk of long COVID symptoms. As compared to participants that completed their primary series of COVID-19 vaccination (receiving 2 doses), receiving at least three doses of COVID-19 vaccine were associated with lower odds of reporting any long COVID symptoms. Such findings were consistent with those observed by previous studies [31,32]. Our study suggested that receiving three doses of mRNA (Pfizer-BioNTech) and inactivated (SinoVac CoronaVac) vaccines, the most common choices of COVID-19 vaccines in Hong Kong, were both associated with significantly lower odds of reporting any long COVID symptoms. Moreover, the protective effects conferred by three doses of mRNA vaccines and three doses of inactivated vaccines were similar in reducing odds of long COVID symptoms. These findings supported the growing research that using mRNA COVID-19 vaccine as a booster dose had a protective effect against long COVID symptoms [31] and added new evidence that using inactivated vaccines could also reduce the odds of long COVID symptoms. Therefore, it is important to strengthen the coverage of COVID-19 vaccination booster doses, even in the post-pandemic era. Previous systematic review and meta-analysis revealed that heterologous vaccination (the combination of inactivated-mRNA vaccines) had the highest antibody responses, and it was more effective than homologous vaccination [33]. However, we were not able to observe similar associations in this study due to the relatively small number of participants with heterologous vaccination.

In line with previous studies, older age and female gender were associated with higher odds of having self-reported long COVID symptoms [27,34,35]. Increased age was associated with a decline in organ functions and a slower ability to recover from acute SARS-CoV-2 infection [27]. Previous studies suggested that hormones might play a role in perpetuating the hyper-inflammatory status of the acute phase after recovery [35,36]. As compared to males, females produced stronger IgG antibodies in the early phase of COVID-19, which might also play a role in perpetuating disease manifestations [35,36]. In addition, females are likely to be more attentive to their body than males [34]. As compared to asymptomatic infection, participants reported having moderate or severe symptoms of acute SARS-CoV-2 infection had higher odds of reporting long COVID symptoms. Previous studies suggested that in acute infection, the levels of cGAS, STING, and IFN-α were higher in patients with severe symptoms than those with mild or no symptoms [37]. Long COVID symptoms were associated with elevated cGAS, STING, and IFN-α levels [37]. Therefore, individuals affected by SARS-CoV-2 who are older, female gender, and having more severe symptoms should be identified and involved early in the follow-up programmes for long COVID management.

Nonetheless, this study had several limitations. First, it is possible that people without any long COVID symptoms were less likely to be interested in participating in the survey. Therefore, the prevalence of long COVID symptoms was likely to be overestimated by this study. Second, failure to measure the time since the last vaccination was another major limitation of this study. The protection conferred by primary and booster doses of COVID-19 vaccines were declining over time [38]. Hong Kong started to offer a third dose of COVID-19 vaccine as booster on 11 November 2021 [39]. The fifth wave of COVID-19 outbreak in Hong Kong occurred between December 2021 and April 2022. Therefore, the variance of time interval between the completion of booster dose and confirmed SARS-CoV-2 infection during the fifth wave might be relatively small (0–5 months) in this study. Third, as compared to the census data in Hong Kong, older adults were under-sampled by this study [29]. Our findings mainly reflected the situation of younger adults in Hong Kong. Fourth, the data were self-reported and recall bias might exist. Information about COVID-19 vaccination status and long COVID symptoms could not be verified. Fifth, we were not able to know the response rate of this study due to the sampling design. We could not collect information from those who have seen the study information but did not access the link of online survey. Characteristics between the refusals and participants might be different, selection bias existed. Moreover, the causal relationship between the COVID-19 vaccination status and presence of long COVID symptoms could not be established as this was a cross-sectional survey study.

## Conclusions

Among people with a history of SARS-CoV-2 infection in Hong Kong, 40.9% and 16.1% self-reported having any and multiple (≥3 types) long COVID symptoms. Individuals affected by SARS-CoV-2 that are older, female gender, and having more severe symptoms should be identified and involved early in the follow-up programmes for long COVID management. Our findings also highlighted the role of COVID-19 vaccination booster dose in reducing the risk of long COVID symptoms. It is important to strengthen the coverage of COVID-19 vaccination booster doses, even in the post-pandemic era.

## Supporting information

Zheng et al. supplementary material Zheng et al. supplementary material

## Data Availability

The data presented in this study are available from the corresponding author upon request. The data are not publicly available as they contain sensitive personal behaviours.

## Supplementary material

For supplementary material accompanying this paper visit http://doi.org/10.1017/S0950268824001687. click here to view supplementary material
