# Supplementary material for: Receiving three doses of inactivated or mRNA COVID-19 vaccines was associated with lower odds of long COVID symptoms among people with a history of SARS-CoV-2 infection in Hong Kong, China: a cross-sectional survey study
Source: Epidemiol Infect. 2024 Dec 11;152:e166. doi: 10.1017/S0950268824001687 (PMC11696583; doi:10.1017/S0950268824001687)
Supplement: Zheng et al. supplementary material [file S0950268824001687sup001.docx]

Receiving three doses of inactivated or mRNA COVID-19 vaccines was associated with lower odds of long COVID symptoms among people with a history of SARS-CoV-2 infection in Hong Kong, China: A cross-sectional survey study

Chen Zheng ^1,†^, Fuk-yuen Yu ^2,†^, Paul Shing-fong Chan ^2,†^, Fenghua Sun ^1^, Xiang-Ke Chen ^3^, Wendy Ya-Jun Huang ^4, 5^, Stephen Heung-Sang Wong ^6^, Yuan Fang ^1^, Zixin Wang ^2,*^

1. Department of Health and Physical Education, Faculty of Liberal Arts and Social Sciences, The Education University of Hong Kong, Hong Kong, China

2. Centre for Health Behaviours Research, JC School of Public Health and Primary Care, The Chinese University of Hong Kong, Hong Kong, China

3. Division of Life Science, School of Science, The Hong Kong University of Science and Technology, Hong Kong, China

4. Academy of Wellness and Human Development, Hong Kong Baptist University, Hong Kong, China

5. Dr Stephen Hui Research Centre for Physical Recreation and Wellness, Hong Kong Baptist University, Hong Kong, China

6. Department of Sports Science and Physical Education, Faculty of Education, The Chinese University of Hong Kong, Hong Kong, China

† These authors contributed equally to this manuscript

* Corresponding to Dr. Zixin Wang

Room 508, School of Public Health, Prince of Wales Hospital, Shatin, N.T., Hong Kong

Email: wangzx@cuhk.edu.hk

Appendix 1

Table S1 Doses and types of COVID-19 vaccines received by the participants prior to the fifth wave of COVID-19 outbreak in Hong Kong (n=1542)

|  | n | % |
| --- | --- | --- |
| 0 doses | 82 | 5.3 |
| 1 dose of Pfizer-BioNTech | 54 | 3.5 |
| 1 dose of SinoVac | 26 | 1.7 |
| 1 dose of other vaccine | 1 | 0.1 |
| 2 doses of Pfizer-BioNTech | 757 | 49.1 |
| 2 doses of SinoVac | 130 | 8.4 |
| 1 dose of SinoVac + 1 dose of Pfizer-BioNTech | 1 | 0.1 |
| 1 dose of other vaccine + 1 dose of SinoVac | 1 | 0.1 |
| 3 doses of Pfizer-BioNTech | 345 | 22.4 |
| 3 doses of SinoVac | 98 | 6.4 |
| 2 doses of SinoVac + 1 dose of Pfizer-BioNTech | 32 | 2.1 |
| 2 doses of Pfizer-BioNTech + 1 dose of SinoVac | 11 | 0.7 |
| 1 dose of SinoVac + 2 doses of Pfizer-BioNTech | 1 | 0.1 |
| 4 doses of SinoVac | 3 | 0.2 |

Table S2 Associations between types of COVID-19 vaccines received by the participants and self-reported long COVID symptoms (n=1536) ^1^

|  | n/N | OR (95%CI) | P values | AOR (95%CI) | P values |
| --- | --- | --- | --- | --- | --- |
| **1. Self-reported having any long COVID symptoms** |  |  |  |  |  |
| Types of COVID-19 vaccines received by the participants |  |  |  |  |  |
| 2 doses of BioNTech | 331/757 | 1.0 |  | 1.0 |  |
| 2 doses of SinoVac | 56/130 | 0.97  (0.67-1.42) | 0.89 | 0.89  (0.59-1.32) | 0.55 |
| 3 doses of BioNTech | 118/345 | 0.67  (0.51-0.87) | 0.003 | 0.68  (0.52-0.90) | 0.006 |
| 3 doses of SinoVac | 33/98 | 0.65  (0.42-1.02) | 0.06 | 0.57  (0.35-0.90) | 0.02 |
| Mixing doses of SinoVac and BioNTech | 19/44 | 0.98  (0.53-1.81) | 0.94 | 0.98  (0.52-1.85) | 0.95 |
| Not completing primary vaccination series | 73/162 | 1.06  (0.75-1.49) | 0.76 | 0.96  (0.67-1.36) | 0.81 |
|  |  |  |  |  |  |
| **2. Self-reported having ≥3 types of long COVID symptoms** |  |  |  |  |  |
| 2 doses of BioNTech | 124/757 | 1.0 |  | 1.0 |  |
| 2 doses of SinoVac | 26/130 | 1.28  (0.80-2.04) | 0.31 | 1.09  (0.66-1.81) | 0.75 |
| 3 doses of BioNTech | 45/345 | 0.77  (0.53-1.11) | 0.16 | 0.80  (0.55-1.18) | 0.26 |
| 3 doses of SinoVac | 13/98 | 0.78  (0.42-1.44) | 0.43 | 0.64  (0.34-1.24) | 0.19 |
| Mixing doses of SinoVac and BioNTech | 4/44 | 0.51  (0.18-1.45) | 0.21 | 0.47  (0.16-1.40) | 0.18 |
| Not completing primary vaccination series | 36/162 | 1.46  (0.96-2.21) | 0.08 | 1.29  (0.84-1.99) | 0.25 |

^1^ Six participants were excluded from the analysis, including three participants who received 4 doses of SinoVac, one participant who received other type of vaccine, one participant who received 1 dose of SinoVac and 1 dose of other type of vaccine, and one participant who received 1 dose of SinoVac and 1 dose of Pfizer-BioNTech

AOR: adjusted odds ratios, odds ratios adjusted for variables related to sociodemographic characteristics, health conditions and lifestyles, and COVID-19 infection during the fifth wave of COVID-19 outbreak in Hong Kong listed in Table 2

CI: confidence interval
